# Estudio ecológico de la sífilis gestacional y congénita en Colombia, 2012-2018

**DOI:** 10.15649/cuidarte.2326

**Published:** 2022-08-28

**Authors:** Carolina Becerra-Arias, Jorge Luis Alvarado-Socarras, Edgar Fabian Manrique-Hernandez, Jhondrisson Alexis Caballero-Carvajal

**Affiliations:** 1 Secretaría de Salud y Ambiente de Bucaramanga, Colombia. Email: caroba23@hotmail.com Secretaría de Salud y Ambiente de Bucaramanga Colombia caroba23@hotmail.com; 2 Fundación Cardiovascular de Colombia, Colombia. Email: jorgealso2@yahoo.com Fundación Cardiovascular de Colombia Colombia jorgealso2@yahoo.com; 3 Departamento de Salud Pública, Escuela de Medicina, Universidad Industrial de Santander, Bucaramanga, Colombia. Email: fabianmh1993@gmail.com Universidad Industrial de Santander Departamento de Salud Pública Escuela de Medicina Universidad Industrial de Santander Bucaramanga Colombia fabianmh1993@gmail.com; 4 Universidad Industrial de Santander, Bucaramanga, Colombia. Email: jhonalecc22@outlook.com Universidad Industrial de Santander Universidad Industrial de Santander Bucaramanga Colombia jhonalecc22@outlook.com

**Keywords:** Sífilis Congénita, Edad Gestacional, Atención Pre natal, Monitoreo Epidemiológico, Syphilis, Congenital, Gestational Age, Prenatal Care, Epidemiological Monitoring, Sífilis Congenita, Idade Gestacional, Cuidado Pré-Natal, Monitoramento Epidemiológico

## Abstract

**Objetivos:**

Describir el comportamiento de la sífilis gestacional y congénita en Colombia, entre el 2012 y 2018, a partir de registro de notificación Nacional.

**Materiales y Métodos:**

Estudio ecológi co, exploratorio a partir de Notificaciones al sistema de vigilancia de salud Pública. Se estimaron la tasa de incidencia y la razón de prevalencia para cada departamento. Se establecieron cada una las estimaciones según rangos, para los 33 departamentos eva luados y se expresaron en mapas a escala de grises según tasas y razones evaluadas. Además, se presentan curvas epidemiológi cas por semanas notificación para sífilis gestacional y congénita.

**Resultados:**

Arauca, Santander, Cesar y Caldas, presentaron el mayor incremento entre 2012 y 2018 para sífilis gestacional. Para el mismo periodo, Santander, Casanare y Amazonas presentaron un aumento para sífilis Congénita, mientras que en los demás de partamentos se evidenció una disminución en los eventos. Se en contraron diferencias significativas en el reporte de casos entre un año y otro, para el país, en ambos eventos (p< 0,001).

**Conclusiones:**

En Colombia se encontró un aumento de sífilis gestacional, mientras, para sífilis congénita existió variabilidad con tendencia a aumentar en los últimos años.

## Abstract (en)

**Objective:**

To describe the behavior of pregnancy and congenital syphilis in Colombia between 2012 and 2018 according to national notifications records.

**Materials and Methods:**

An ecological exploratory study was conducted based on notifications to the public health surveillance system. The incidence rate and prevalence ratio were estimated for each department. Each estimate was established per ranges for 33 departments evaluated and expressed in grayscale maps based on rates and ratios evaluated. Epidemic curves by week of notification for pregnancy and congenital syphilis are also shown.

**Results:**

Arauca, Santander, Cesar and Caldas had the highest increase in pregnancy syphilis between 2012 and 2018 while Santander, Casanare and Amazonas had an increase in congenital syphilis during the same period. Other departments had a decrease in these events. Significant differences were found in case reporting from year to year in both events in the country (p<0.001).

**Conclusions:**

In Colombia, an increase in pregnancy syphilis was found while a variability with an increasing trend was found for congenital syphilis in recent years.

## Resumo

**Objetivos:**

Descrever o comportamento da sífilis gestacional e congenita na Colombia entre 2012 e 2018, com base nos registros nacionais de notificado.

**Materiais e Método:**

Estudo ecológico, exploratório, baseado em notificares ao sistema de vigilancia sanitária pública. A taxa de incidencia e a taxa de prevalencia foram estimadas para cada departamento. As estimativas foram estabelecidas de acordo com intervalos para os 33 departamentos avaliados e expressas em mapas em escala de cinza, de acordo com as taxas e rácios avaliados. Além disso, curvas epidemiológicas por semanas de notificado sao apresentadas para sífilis gestacional e congenita.

**Resultados:**

Arauca, Santander, Cesar e Caldas apresentaram o maior aumento entre 2012 e 2018 para a sífilis gestacional. No mesmo período, Santander, Casanare e Amazonas mostraram um aumento para a sífilis congenita, enquanto os outros departamentos mostraram uma diminuido nos eventos. Foram encontradas diferengas significativas no relato de casos de um ano para o outro, para o país, em ambos os eventos (p<0,001).

**Conclusóes:**

Na Colombia, houve um aumento da sífilis gestacional, enquanto para a sífilis congenita houve variabilidade com tendencia a aumentar nos últimos anos.

## Introducción

La Organización Mundial de la Salud (OMS) estima que alrededor de 1,3 a 2 millones de embarazos en el mundo son afectados por sífilis gestacional (SG), a pesar de políticas de salud pública, existencia de intervenciones costo efectivas como es el tamizaje gestacional y el tratamiento, una vez se detectan los casos^1^. La SG se ha relacionado con eventos perinatales adversos como aborto, muerte fetal y sífilis congénita (SC), esta última con riesgo de prematurez, bajo peso al nacer, muerte neonatal y discapacidad^2^. La región de las Américas y el Caribe reporta la tercera mayor prevalencia a nivel mundial con un 0,42%, después de África (1,68%) y la región mediterránea oriental (0,57%)^1^^,^^3^. Debido a lo anterior, actualmente la sífilis continúa como problema de salud pública, con mayor impacto en la población más vulnerable y de estratos socioeconómicos bajos^3^.

Por lo anotado, continúa siendo una condición objeto de estrategias e intervenciones en países de la región, posiblemente por casos sub-diagnosticados, e incluso algunos no tratados apropiadamente^4^. Sumado a lo anterior, hoy es una patología reemergente en algunos países como en Brasil, lo que determina evaluar la situación en Latino América^5^. Es por esto que las estrategias deben ser evaluadas de formas dinámicas, con el fin de adaptarse a nuevos escenarios como población desplazada, migrante o en la pobreza, para así lograr la reducción que se plantea en metas de orden internacional. Una de estas últimas es la reducción de SC a 0,5 casos por cada 1.000 nacidos vivos o menos en la región de Latino América y el Caribe. Para lograr esto es necesario una disminución de la incidencia de sífilis global, tamización durante la gestación, tratamiento oportuno, lo que debería generar un impacto en la salud neonatal. Hoy la SG es responsable de aproximadamente 300.000 muertes fetales y neonatales por año, y adicionalmente unos 215.000 recién nacidos con riesgo de muerte neonatal temprana^6^.

Con respecto a la estimación nacional de SC, Colombia para el 2020 presentó una incidencia de 1,2 casos por 1.000 nacidos vivos, según boletín epidemiológico, semana 30 de 2020 del Instituto Nacional de Salud. En adición, se ha observado un incremento en las estimaciones de SG y SC en los últimos 5 años, lo que podría tener relación con la implementación de la nueva guía de práctica clínica a partir del 2015^8^. Pese a esto, no se descarta a su vez el aumento producto del robustecimiento de actividades de búsqueda de casos sugestivos, dentro del marco de las atenciones primarias en salud.

Estos datos en conjunto, han motivado un plan de intensificación para la eliminación de la sífilis congénita, debido que las estrategias previamente establecidas no lograron la reducción de este evento en la magnitud prevista. Para optimizar tales estrategias y determinar prioridades de atención, es importante identificar las tendencias en cada país, delimitadas por regiones e incluso por municipios. Al mismo tiempo, esto permitiría evaluar las variables poblacionales que se relacionarían con un potencial aumento/disminución en el número de casos, así como características propias de la población, el entorno, la cultura, y demás que pudieran intervenirse. De esta forma, podría concebirse, además del alcance de los objetivos propuestos en cada gobierno, su impacto sobre el estado de salud en el binomio madre - hijo.

Por lo anterior, a pesar de innumerables estrategias existentes, la sífilis congénita y gestacional no exhibe tendencias al decremento, como se esperaría, por lo que es clara la necesidad de plantear intervenciones adicionales y evaluar las actuales a nivel regional. Es posible que mejorar la calidad de los controles prenatales (CPN) sea un punto a trabajar a corto plazo. Revisiones previas en Colombia han demostrado que existe una percepción de mala calidad hacia estoscontroles; sumado a esto otros factores como los bajos ingresos económicos y la educación están relacionados con una mala adherencia a los CPN^7^. Esto hace presumir que algunos factores de inequidad, como el acceso a los servicios de salud y las condiciones sociales, podrían tener una carga importante como principales limitantes que dificultan alcanzar los objetivos establecidos.

El objetivo del presente estudio es describir el comportamiento de la sífilis gestacional y congénita en Colombia, durante el periodo comprendido entre 2012 y 2018, a partir de registros de notificación al Sistema de Vigilancia en Salud Pública del país. Con las conclusiones e hipótesis aquí planteadas, se podrán plantear diseños más amplios y robustos, que permitan identificar los factores relacionados con el desenlace de estos eventos, a fin de ir en la misma vía que las disposiciones de orden internacional y nacional.

## Materiales y Métodos

Estudio ecológico, exploratorio y de fuente secundaria, a partir de notificaciones al Sistema de Vigilancia en Salud Pública (SIVIGILA) de Colombia, correspondiente al periodo 2012-2018. Los archivos se obtuvieron por cada semana epidemiológica en cada año y departamento, en la página del Instituto Nacional de Salud (INS), vigilancia rutinaria histórica, del portal SIVIGILA. Adicionalmente, se consultaron los registros de nacidos vivos (NV) y de mortinatos, disponibles de 2012 a 2018, en la página del Departamento Administrativo Nacional de Estadística (DANE), por año y departamento.

Se tomaron datos de 32 departamentos con reporte del conteo de número de casos para cada evento, sífilis gestacional (SG) y sífilis congénita (SC), y Bogotá, como distrito capital, para un total de 33 unidades de observación. Asimismo, para Colombia se tomó el total de casos por cada año. Con esto se estimó la razón de prevalencia y la tasa de incidencia, respetivamente, por cada año y departamento del país, a partir de las siguientes fórmulas, según protocolos de vigilancia en salud pública, del INS:




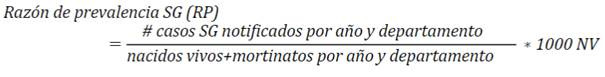







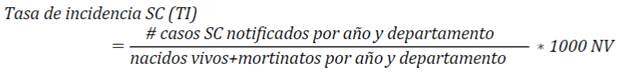




El mapa para la RP y la TI se construyó a partir de las estimaciones por departamento en cada año. Se realizó el diseño de los mapas cloropléticos en escala de grises, para lo cual se usó Adobe Ilustrator, con 5 puntos de corte para los valores de los estimadores en cada evento. Se graficó el estimador por evento y año según entidad territorial (ET), cada una correspondiente a un departamento, para evidenciar visualmente cambio en las tendencias. A cada ET se les asignó un número, correspondiente a un color dentro de la escala, según el rango en el que se encontrara por año. Del mismo modo, se exploró la distribución del número de casos reportado en cada ET, así como para el total correspondiente al país, a fin de identificar asimetrías en esta variable. Se evaluaron diferencias en el número de casos notificados entre un año y otro, mediante el análisis de varianza (ANOVA), tanto para SG como para SC, con el reporte del valor *p* en cada caso. Se consideró estadísticamente significativo un valor *p*<0,05.

Para la elaboración de las curvas epidémicas se constituyó una base de datos en Excel^(®)^, que contenía el número de casos y la tasa, para Colombia, correspondiente al total de notificaciones en cada año. Para los años 2012 a 2018 se tomaron las 52 semanas epidemiológicas. De esta forma, para el numerador se tomó el número de casos notificados para cada semana, mientras que en el denominador se identificó a la población correspondiente al año en cuestión, en el país. Para el caso de SC se identificó el total de NV+mortinatos, mientras que en la SG este correspondió al total de mujeres en edad fértil (EF) (15 a 49 años) consultadas en visor del DANE.

Se tomaron los datos de cobertura de CPN por departamento, de los indicadores “Así vamos en salud”, disponibles de forma libre vía web, para revisar la variación por quinquenio entre 2005 y 2015, y generar posibles hipótesis con relación a los hallazgos de SG y SC, se guardaron los hallazgos tabulados y reportados en una base de datos como en un repositorio público (Zenodo/Data-set)^8^ guardando la confidencialidad de los participantes del estudio.

## Resultados

En la Gráfica 1, el año del periodo evaluado con la mayor tasa de SG fue el 2018, seguido por el 2017, lo cual sugiere que este evento ha presentado un incremento, con el transcurrir de los años. En el último año, (2018) las semanas con mayor notificación corresponden a la 36, 21 y 30. Durante la semana 44 en 2019 se identifica un incremento del 31,3% en la TI, respecto a 2017 y 50% comparado con 2016. Para la semana 52 se identifica un marcado ascenso en 2016 (respecto a los otros años), con un pico equivalente a una tasa de 2,6 x 100.000 mujeres EF, aportado principalmente por Antioquia y Bolívar, con 49 y 51 casos, respectivamente. En contraposición a lo mencionado antes, el 2014 presentó las tasas más bajas con 0,06 x 100.000 mujeres EF en la semana 1, la más baja de todo el periodo analizado, lo cual resultó en un 23,7% menos al compararse con 2013, y 37,7% menos respecto al 2015. El número de notificaciones entre un año y otro resultó diferente estadísticamente (*p*<0,001), específicamente para los años 2014, 2017 y 2018, con respecto a los demás años evaluados.

**Gráfica 1:**
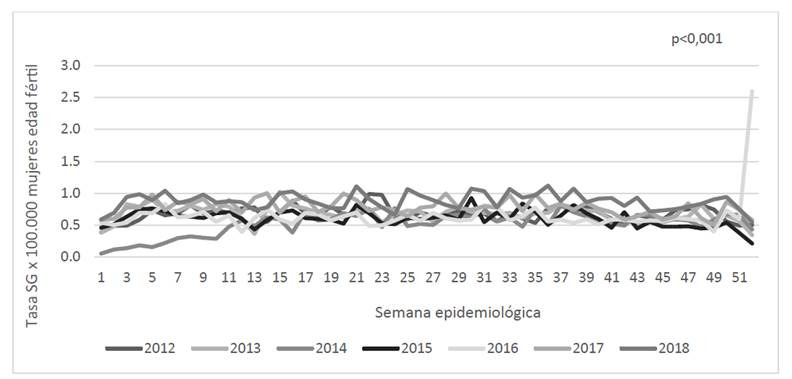
Curvas epidémicas Sífilis Gestacional 2012 a 2018, según semana epidemiológica

**Gráfica 2:**
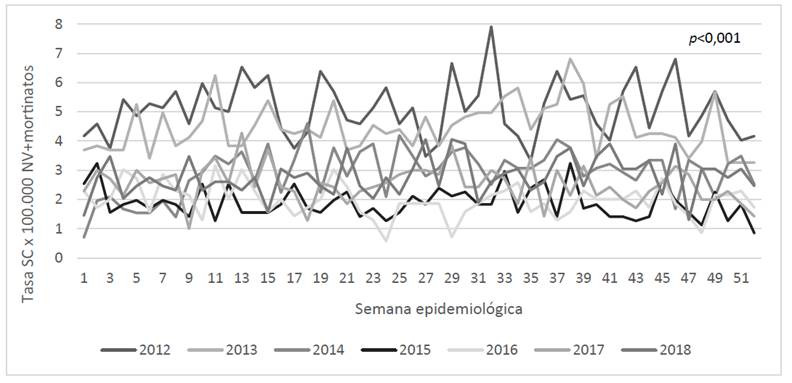
Curvas epidémicas Sífilis Congénita 2012 a 2018, según semana epidemiológica

En el análisis de eventos por SC se observa en la Gráfica 2, que del periodo evaluado (2012-2018), el 2012 resultó tener las mayores tasas, con valores más elevados en las semanas 32, 46, 29, 43 y 13 (7,9; 6,8; 6,7 y 6,5 x 100.000 NV+mortinatos, respectivamente). Valle, Bogotá, Antioquia y Bolívar reportaron el mayor número de casos en este año, mientras que Guainía, Guaviare, Vichada y San Andrés notificaron el número de casos más bajo, con respecto a los demás departamentos. Los años 2015 y 2016 aportaron casos en menor medida, con las tasas más bajas reportadas en las semanas 24 y 29 en 2016. Sumado a lo anterior, se encontraron diferencias estadísticamente significativas en el número de casos notificados por año (*p*<0,001) para SC, lo cual se soporta con lo presentado en la gráfica, en la que se evidencia mayor dispersión entre una curva y otra, en contraste con lo hallado para SG. Se encontraron diferencias estadísticamente significativas entre un año y otro, excepto entre 2015-2016, 2014-2017, 2014-2018 y 2017-2018.

**Figura 1:**
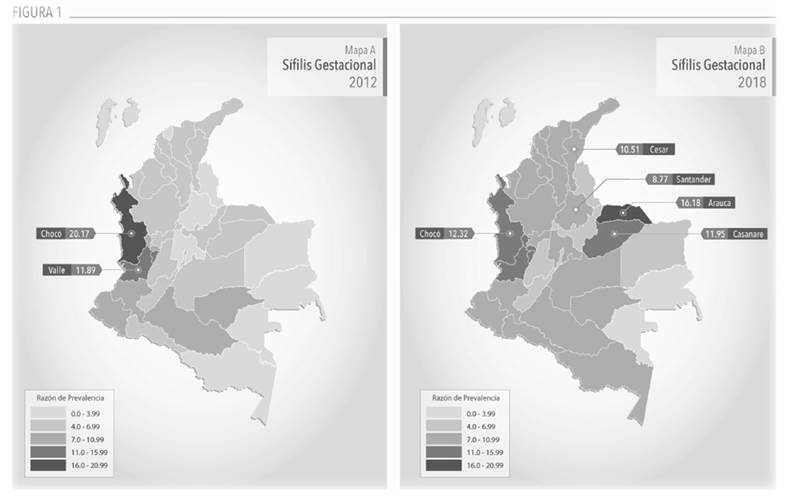
Mapas Coropléticos Razón de Prevalencia Sífilis Gestacional, por departamento, 2012 y 2018

Con respecto a los estimadores según departamento, el mapa de SG (Figura 1) permite identificar los cambios que han tenido las ET en la tendencia del evento, en el periodo evaluado. Uno de los más marcados fue el de Arauca, que pasó de una RP de 4,7 a 16,2 x 1000 NV. Este estimador con un crecimiento del 244% lo convierte, a su vez, en el más alto del 2018. Situación similar se encontró para Santander, Cesar y Caldas, en los que la razón se aumentó más de 4 unidades, en cada caso. Por otro lado, Chocó fue el único departamento que evidenció una disminución del 39,4%en la RP respecto a 2012, de 20,2 a 12,3 x 1000 NV.

**Figura 2:**
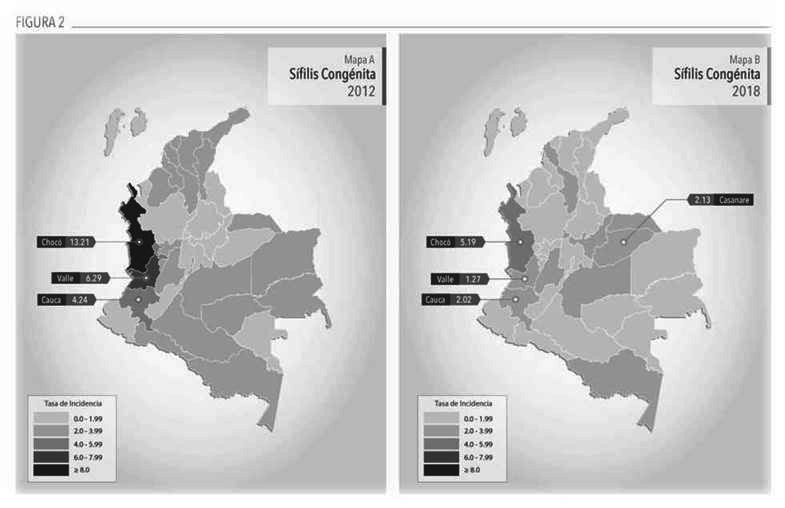
Mapas Coropléticos Tasa de Incidencia Sífilis Congénita, por departamento, 2012 y 2018

Por otro lado, el mapa de SC (Figura 2) muestra una disminución en las tasas de incidencia en el país, durante el periodo evaluado, excepto Santander (de 0,7 a 1,2 x 1000 NV), Casanare y Amazonas, en los que se evidenció un incremento. La mayor reducción en el estimador la presentó Chocó con un 60,6%(de 13.2 a 5.2 x 1000 NV), seguido por Arauca, y Amazonas (3,6 y 2,9 x 1000 NV). Sin embargo, Chocó persiste con una de las tasas más altas del país.

**Tabla 1:** Razón de Prevalencia y Tasa de Incidencia de SG-SC en Colombia, por año

Año	SG (RP x 1000 NV)	SC (TI x 1000 NV)
2012	6,02	2,65
2013	6,23	2,33
2014	4,84	1,55
2015	5,57	0,98
2016	6,16	1,03
2017	7,36	1.31
2018	8,49	1,45

SG: sífilis gestacional, SC: sífilis congénita, RP: Razón de Prevalencia, TI: Tasa de Incidencia

La tendencia de los estimadores generales para Colombia, como es de esperarse, se asemeja a la de los departamentos. Para SG hay un aumento con el paso de los años, a partir del 2014, tras un descenso entre 2013 y 2014 (Tabla 1). De otro modo, la SC exhibió decrementos en el tiempo hasta 2015; a partir de allí se presentan aumentos sustanciales hasta 2018.

Respecto a otra variable que podría estar relacionada con los eventos, se presenta en la Gráfica 3 el porcentaje en la cobertura del CPN, según identificación por departamento, para los quinquenios 2005, 2010 y 2015. En primer lugar, se evidencia el logro alcanzado en algunas entidades territoriales, respecto a la cobertura cercana a 100%, adicionalmente mantenida a lo largo del tiempo. Tal es el caso de Quindío, Risaralda, San Andrés, Norte de Santander y Santander. En segundo lugar, deja al descubierto las brechas que podrían existir en Vaupés, Chocó, Guainía, La Guajira, Vichada y Amazonas, pues no solo presentan las coberturas más bajas, sino que no se ha logrado incrementar este indicador en los 10 años de medición revisados. Con respecto al logro del CPN en Colombia, en 2005 pasó de 93,7% a 97% en 2010, y finalmente a 97,5% en 2015; pese a que se ha incrementado de forma general, las acciones deben orientarse según características y necesidades propias de cada región. En concordancia con lo expuesto, al revisar las RP de SG para el 2015 se identificó a Chocó, el cual presentó además de una de las razones más altas de ese año, la segunda menor cobertura de CPN.

**Gráfica 3:**
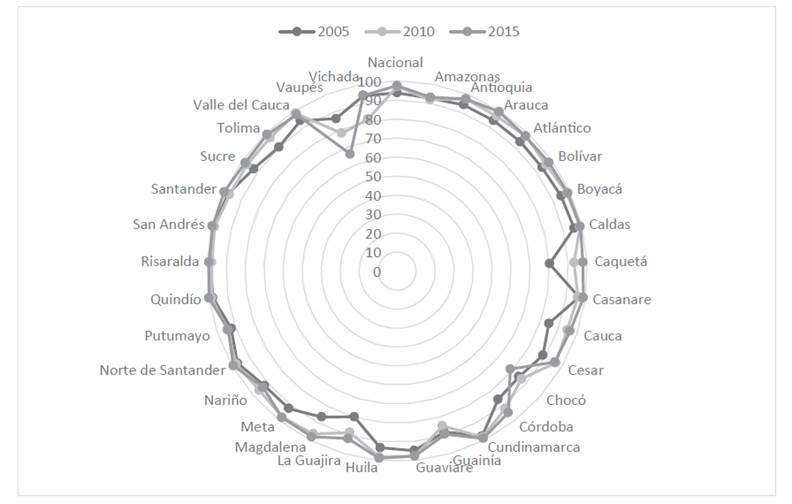
% Cobertura CPN por quinquenio y departamento

## Discusión

A partir de los hallazgos del presente estudio se identifica una tendencia hacia el aumento de SG. Se evidencian algunas regiones críticas, como Arauca que mostró la RP más alta para el 2018, seguido por el departamento del Valle y el Chocó (12,9 y 12,3x1.000 NV, respectivamente); sin embargo, este último con mejoría en los últimos años. No se conocen estrategias adicionales a las dispuestas desde el orden nacional, para orientar las acciones en salud pública en Chocó u otro departamento para SG y SC. La posible explicación a la disminución en el departamento antes mencionado, podría ser el subregistro o problemas relacionados con la notificación de casos al sistema de vigilancia, dado que la tendencia nacional de SG en el país indica un decremento hasta 2014, y a partir de allí un incremento. Lo anterior a su vez, podría relacionarse con ausencia de personal en el área de vigilancia epidemiológica, de forma permanente, o falta de capacitación al personal de salud de las instituciones de salud para identificar los casos que cumplan con la definición del evento, entre otras cosas por la alta rotación del mismo.

Lo antes expuesto indicaría que, para el caso de Chocó, pese a presentar un número absoluto de casos notificados con tendencia secular, al estimar la RP, tomando en consideración los nacidos vivos y mortinatos, este indicador da como resultado el tercer valor más alto de Colombia. Es así que, para efectos de análisis del evento, como punto de partida para estrategias en salud pública, resulta fundamental considerar este indicador, para abordar la población clave a la que se dirigirían dichas acciones. Para adicionar a lo enunciado, según boletín epidemiológico de la semana 30 de 2020, emitido por el Instituto Nacional de Salud (INS), a dicha fecha solo en cuatro regiones se observó una disminución de casos acumulado de SG menor al esperado; estos correspondieron a Bolívar, Boyacá, Risaralda y Valle^9^. Estos hallazgos podrían estar relacionados con el nivel socioeconómico de las regiones; es decir, mayores estimadores presentes en áreas con mayor pobreza^1^^,^^5^^,^^9^.

El impacto global de la SG y SC trasciende a una problemática de salud pública en América Latina, relacionado con determinantes sociales relevantes. Haití por ejemplo, registra uno de los índices más altos del continente, incluso con tendencia al aumento en los últimos años, similar a nuestros hallazgos. Se conoce que durante los controles prenatales un 7,1% de gestantes son detectadas tardíamente para SG, con diagnósticos entre la semana 23 y 25, con la posterior asociación a eventos adversos incluso tras el nacimiento^10^.

Uno de los puntos a resaltar con los eventos revisados es que resultan no solo ser una limitante de países en vía de desarrollo o poco desarrollados. Según cifras del Center for Diseases Control and Prevention (CDC), en Estados Unidos la tendencia de la SG ha venido en aumento desde el 2012, lo cual se incrementa paralelamente con los casos reportados de sífilis primaria y secundaria, en mujeres en edad fértil en ese país^11^. Esto al mismo tiempo se relacionaría con las estimaciones de sífilis en población general en ese país, lo cual se vislumbra con un aumento en la tasa de infección por sífilis en hombres, hasta comprometer el 86% de todos los casos en Estados Unidos (EU)para el 2018. Existe también una estrecha relación con la infección por VIH y otras infecciones de trasmisión sexual^12^.

Por otro lado, la existencia de métodos de diagnóstico y tratamiento efectivos sigue siendo la principal herramienta para contrarrestar la diseminación de la sífilis en todos los grupos etarios y lugares; sin embargo, las fluctuaciones en los indicadores y estimaciones en SG y SC aún ameritan revisiones detalladas, para tratar de concebir en conjunto las potenciales limitaciones que imposibilitan el alcance de los logros esperados. El comportamiento de la SG ha permanecido estable desde el 2009 en el panorama mundial, excepto en Brasil en el que la tendencia ha presentado incrementos con el tiempo^4^. Reportes recientes para este país documentan un aumento en SG del 202% entre el año 2010 y 2015^13^. A partir de reportes para el 2015, las mayores tasas de SC (x 1000 NV) se reportaron en Brasil (564,1), seguido por Colombia (521.9), lo que reflejaría la existencia de condicionantes regionales en estos países, que favorecerían el incremento de los eventos^14^.

Como reflejo de lo anterior podría encontrarse un mayor grado de vulnerabilidad en las poblaciones de esos países, dado entre otras cosas por disparidad socioeconómica y las barreras de acceso a servicio de salud^12^^,^^15^^).^ El acceso a estos últimos son de vital importancia para logra detectar casos y tratar de forma oportuna las gestantes con SG. Una de las actividades dispuestas es la realización del control prenatal de calidad que logre impactar favorablemente los indicadores. No obstante, reportes recientes de la OMS en el 2018 indican que en Colombia el 62,7% de las gestantes fueron tamizadas para SG en las consultas prenatales, comparadas con Ecuador (78,1%), Brasil (88,2%), Argentina (90,8%), Perú (94,3%), Bolivia (96%) y Chile (100%). Lo que claramente demuestra una brecha entre países y regiones en un mismo continente. Se determinan así puntos a mejorar, como disminuir la variación del CPN, que oscila entre 40,7% y 85,4% en el 2016 y 2010 respectivamente^13^^,^^16^^).^

Acerca de este tópico, el CPN en Colombia es un programa obligatorio e independiente del sistema de seguridad social. Su ejecución pretende, además del tamizaje para SG, detectar otras morbilidades durante esta etapa de la mujer, con el propósito de aminorar la morbilidad materna extrema, la mortalidad materna, la mortalidad perinatal y los desenlaces desfavorables como la SC, entre otros. Las posibles aristas del no cumplimiento de un porcentaje mínimo de CPN en las gestantes recaerían en condiciones de vulnerabilidad social. Tal podría ser el caso de inconvenientes económicos para el transporte o acceso a servicios de salud, lo cual impediría lograr el número de controles óptimos establecidos en Colombia, mínimo 4 durante la gestación^17^.

Con respecto al diagnóstico de SG, a partir de 2013 se estableció que se debe realizar mediante pruebas rápidas y tratamiento de sífilis en la primera consulta prenatal, con el objetivo de lograr la disminución de casos de SC. Sumado a esa directriz es fundamental el acompañamiento del personal de salud en este proceso, el cual debe estar debidamente capacitado y actualizado, lo cual fortalece los programas instaurados desde el orden territorial. En consideración a lo anterior, previamente se ha descrito una deficiencia en el conocimiento del manejo de la sífilis gestacional entre el personal de salud, incluidos médicos. Esto último podría ir de la mano con el nivel de confianza de la comunidad hacia el personal de salud^18^.

Esto no es exclusivo de países como Colombia, se ha encontrado cierta alteración en la calidad de la atención en Estados Unidos como la falta de tratamiento materno adecuado a pesar del diagnóstico oportuno de sífilis durante el embarazo (30,7%), seguida de cerca por la falta de atención prenatal oportuna (28,2%). Pero, además las dificultades no son las mismas en todas regiones. Resulta relevante considerar el momento del diagnóstico durante la gestación, dado que puede ser una variable de las posibles demoras para el mismo, y por ende para el inicio del tratamiento^19^. Si bien el CPN es relevante en cualquier sistema, debido a la multicausalidad relacionada con la SG y SC, la asistencia a los CPN puede no ser suficiente. Por consiguiente, es necesario identificar el número de visitas a los mismos con el fin de reforzar el seguimiento de las maternas, junto con la dispensación de tratamiento a la pareja o compañero sexual. Adicionalmente, considerar la posibilidad de re-infecciones cuando lo anterior no se logra, y porque ha resultado ser un factor determinante en el éxito de la curación de la SG y la prevención de SC^20^. De esta manera, se evidenciaría que para el año 2015, Quindío, Arauca, Risaralda y Casanare lograron una cobertura del CPN de 99% o más, pese a lo cual presentaron RP de SG entre 6,3 y 10,8 x1000 NV. De esta forma, se justificaría mejorar la calidad en los CPN y el abordaje integral de las maternas, además de la cobertura^21^.Por otro lado, en número de casos de SC son menores a los notificados por SG, lo cual es esperable por la detección e implementación de tratamientos en las gestantes. Así mismo, no es posible descartar la presencia de subregistros, lo que podría impactar en el porcentaje del diagnóstico y tratamiento entre las poblaciones, y constituir el punto de partida en la diferencia de estos indicadores^22^. Es claro que la capacitación continua en registros y vigilancia en salud pública son claves para fortalecer la atención, reducir la transmisión vertical y permitir el análisis permanente de estas limitantes^23^.

Como resultado de las diversas estrategias e intervenciones dispuestas, se evalúan los casos de SG y SC. Para el caso del presente estudio se observan fluctuaciones entre un año y otro; para SG los años 2017 y 2018 han presentado mayores tasas, respecto a años previos, mientras que en SC la mayor tasa corresponde a 2012, con disminución posterior y nuevamente incremento en 2017 y 2018. Lo enunciado podría estar relacionado con situaciones especiales, ocurridas al interior del país en los años en mención, como es el desplazamiento, el conflicto armado, la migración, entre otros fenómenos que podrían presentarse^24^.

Alzate y colaboradores, en 2012, evaluaron las disparidades en la incidencia de sífilis congénita en Colombia, de 2005 a 2011. Allí se encontró que en el país la incidencia de SC se proyectaba hacia el aumento, con Antioquia, Bogotá D.C y Valle con el mayor número de casos reportados, lo cual resulta equivalente a lo hallado en el presente trabajo. Es así como se pone en evidencia la brecha de la ocurrencia de SC al interior del territorio colombiano, lo cual podría demostrar los diferentes enfoques en los programas de promoción de la salud y prevención de las transmisión materno-infantil de enfermedades infecciosas, como la sífilis, el VIH, la gonorrea, entre otras infecciones de transmisión sexual, desde las entidades territoriales y departamentales^25^.

El presente estudio posee ventajas como lo es el trabajar con datos agrupados, a partir de datos individuales de notificaciones al SIVIGILA en el país. De esta forma, se incluyen todos los casos reportados al ente nacional, de aquellos captados por el sistema de salud, según el cumplimiento de definición de caso en cada evento. Algo similar ocurre con los datos de NV + mortinatos, recolectados y publicados por el DANE, ente central y regulador de información demográfica en el país. Asimismo, los estimadores aquí proyectados hacen parte del análisis de información, dispuesto dentro del apartado para este fin, en cada protocolo de vigilancia en salud pública para SG y SC.

Aunque ciertamente presentan ventajas claras, existen puntos que pueden ser reforzados durante el proceso de la notificación, en todas sus etapas. Resulta fundamental reforzar la gestión de vigilancia en salud pública, pues la mayor proporción de casos de SG y SC notificados corresponden a vigilancia pasiva. De esta forma, promover la Búsqueda Activa Institucional (BAI) al interior de centros de atención a gestantes y maternas facilitaría la identificación de casos probables, a partir de fuentes adicionales de información. Así mismo, las Búsquedas Activas Comunitarias (BAC) serían el puente para captar gestantes sin asistencia a CPN, así como aquellas diagnosticadas y no tratadas, o incluso para quienes las parejas sexuales no han iniciado el tratamiento. Lo anterior bajo los principios de la Atención Primaria en Salud (APS), que puede ejecutarse de forma simultánea con el ejercicio de los lineamientos de vigilancia en salud pública^26^.

Con respecto a las acciones en la comunidad, que podría jugar un rol determinante en la prevención de Infecciones de Transmisión Sexual, incluyendo SG y SC, está la educación y comunicación. Robustecer las medidas de autocuidado personal, los métodos anticonceptivos de barrera, así como el ejercer una sexualidad responsable, son un punto de partida que puede sumarse a la participación social, en el objetivo de lograr un mayor control en estas infecciones^27^. Dentro de esta última, la movilización comunitaria y social de acciones estratégicas, la intersectorialidad a partir de autoridades locales, y la inclusión de líderes comunitarios podrían contribuir con la orientación de actividades que permitan la apropiación de medidas de prevención por parte de las poblaciones más vulnerables, así como la diseminación a través de diferentes medios de comunicación, para lograr llegar a una gran proporción de gestantes y mujeres en edad reproductiva.

Dentro de las limitaciones del presente trabajo debe reconocerse la no disponibilidad de información para otras variables, que deberían evaluarse juntamente con las acá estudiadas, como es el caso del porcentaje de gestantes tamizadas, con tratamiento, junto con el estado de afiliación y acceso al Sistema General de Seguridad Social. Este último factor es indudablemente un determinante de mayor morbimortalidad, en patologías de notificación obligatoria, pues se ha documentado una desigualdad marcada en el régimen subsidiado (más pobres), el cual impacta en la salud sexual y reproductiva, lo que incluye la SG y SC^28^.

Por otro lado, tampoco pueden obviarse los subregistros en el Sistema de Vigilancia en el país, para la notificación de Eventos de Interés en Salud Pública, lo cual podría resultaren estimaciones en SG y SC por debajo a las esperadas. Por ejemplo, en Brasil se encontró una subestimación del 32% en los casos de SG y de 17,4% en SC^13^. Es así como el seguimiento de los datos en los sistemas de Vigilancia en salud pública es indispensable para evaluar el cumplimiento de los objetivos, a medida que se realiza la retroalimentación de la calidad de estos, a las fuentes primarias que generan la información. De no reconocer dicho punto dentro del sistema, este podría constituirse en una limitante para la evaluación de las medidas implementadas, así como para estructurar nuevos programas y su impacto real^29^. Adicionalmente, dada que la fuente de información son los casos reportados en SIVIGILA teniendo en cuenta la definición de caso de SG y SC para cada año, esto puede presentar limitaciones para realizar las comparaciones antes y después del cambio de la definición de caso en Colombia en el 2014. Esta limitación se presentó dado que para el análisis no se tuvo disponibilidad de los registros individual para reclasificar los casos bajo la misma definición como lo realizado en otros estudios^29^; sin embargo, este estudio permite evidenciar el comportamiento de SG y SC en Colombia a pesar de sus limitaciones.

Al considerar conjuntamente las variables no evaluadas podrían tomarse en cuenta indicadores de procesos, como aquellos de la identificación y tamización de gestantes en el país, así como los de resultado (% tratadas y curadas, por ejemplo). Esto podría identificar las actividades a reforzar, con el fin de optimizar los recursos destinados al control de la SG y SC, lo cual se reflejaría finalmente en la disminución de la tasa de estos eventos. A partir de los objetivos dispuestos desde diferentes órdenes, el Plan Decenal de Salud Pública (PDSP 2012-2021), dentro de las metas del componente “Prevención y atención integral en Salud Sexual y Reproductiva”, establece que para el 2021 se pretende alcanzar y mantener la incidencia de sífilis congénita en 0,5 casos o menos, incluidos los mortinatos, por cada 1.000 nacidos vivos^6^. A partir de nuestros resultados, para la TI de 2018 (1,45 x 1.000 NV), se esperaría que, para cumplir con lo esperado, el estimador lograra reducirse en 34,5% entre el último reporte y el correspondiente a 2021. No obstante, dentro del periodo evaluado en ninguno de los años se logró estar por debajo de los 0,98 por 1.000 nacidos vivos, por lo que sería necesario redoblar los esfuerzos del diagnóstico y tratamiento oportunos, así como las actividades de seguimiento, de forma que se aborde integralmente la gestante y sus contactos sexuales.

En este sentido, la Estrategia Global del Sector Salud en ITS definió para el 2020, como parte de las metas para lograr el objetivo, al menos en el 70% de todos los países el tamizaje del 95% de mujeres en embarazo para sífilis, y en el 95% de aquellas seropositivas el tratamiento con mínimo una dosis de Penicilina Benzatínica^6^. Por lo anotado, las metas no se han logrado a la fecha propuesta y las políticas no han sido efectivas, dado que se tiene un rezago en los objetivos para el 2030 con una reducción en la incidencia de sífilis hasta en un 90%, dadas las acciones intensificadas encaminadas desde 2016, en general para las ITS. Es esta la oportunidad de que cada entidad realice la revisión al interior de sus poblaciones, de manera que se logren detallar y tomar en cuenta las características de la misma, para de esta forma aplicar los lineamientos nacionales, según las necesidades y prioridades de cada una, reflejado en servicios basados en el contexto. Existen datos de fallas en el sistema de prevención, donde los principales son atención prenatal inoportuna, inoportunidad para la realización de pruebas para sífilis, tratamientos inadecuados e identificación de seroconversión tardía en el embarazo. Sin embargo, esto puede estar influenciado por la disparidad entre las regiones y factores étnicos, raciales^19^. Por otro lado, la persistencia de sífilis en las regiones estaría relacionado con conflictos armados, lo cual origina fenómenos migratorios, que traen consigo consecuencias a largo plazo, producto de rápida urbanización, como son la pobreza, el trabajo sexual, inaccesibilidad a sistema de salud, lo cual podría estar relacionarse con la persistencia de esta condición patológica^30^.

## Conclusiones

Para concluir, la situación de la SG y SC en Colombia continúa siendo un problema de salud pública. Resulta oportuno y necesario re-evaluar los grupos poblacionales a los que se dirigen las estrategias, con el fin de identificar si en realidad se llega a la población blanco. Tampoco se han alcanzado las metas establecidas para los eventos, pues la TI de SC es superior a los 0,5 casos x 1.000 NV, y según reportes previos, en 2018 no se superó el 70% de mujeres tamizadas para SG. Es evidente que aún existen casos no reportados, por mujeres no diagnosticadas durante la atención prenatal, la cual debería alcanzar a la totalidad de gestantes del país. Mientras tanto, muchos de estos resultarán en SC, incluso en aquellas en las que, tras el diagnóstico, no se cumple con el tratamiento.

Pese al despliegue de iniciativas definidas en planes de índole internacional, contrario a lo esperado, las tendencias de la SG y SC exhiben un incremento de forma general. Esto traería consigo sobrecargas al sistema salud por causas prevenibles, y aumento de la brecha social existente en nuestro país, evidenciada entre los departamentos Con los resultados del presente estudio se lograron identificar los años con las mayores tasas de notificación, para considerar dentro de las hipótesis a evaluar, con diseños posteriores, el efecto del fenómeno de la migración con las barreras de acceso a la atención en salud de estas mujeres, diagnóstico tardío, dificultades para el acceso/adherencia al tratamiento, y los seguimientos que se desprenden de allí. Se requiere un manejo integral con actores directos e indirectos para buscar disminuir las tasas e identificar factores comunes que puedan estar asociados, pero sin una intervención adecuada. Lo anterior, bajo la mirada de aquellos departamentos en los que las variaciones fueron mayores, y que podrían entretejer una dinámica más agudizada de las barreras expuestas.
